# Community-wide promotion of physical activity in middle-aged and older Japanese: a 3-year evaluation of a cluster randomized trial

**DOI:** 10.1186/s12966-015-0242-0

**Published:** 2015-06-23

**Authors:** Masamitsu Kamada, Jun Kitayuguchi, Takafumi Abe, Masataka Taguri, Shigeru Inoue, Yoshiki Ishikawa, Kazuhiro Harada, I-Min Lee, Adrian Bauman, Motohiko Miyachi

**Affiliations:** Department of Health Promotion and Exercise, National Institute of Health and Nutrition, 1-23-1 Toyama, Shinjuku-ku, Tokyo 162-8636 Japan; Division of Preventive Medicine, Brigham & Women’s Hospital, Harvard Medical School, 900 Commonwealth Ave East, Boston, MA 02215 USA; Physical Education and Medicine Research Center UNNAN, 1212-3 Mitoya, Mitoya-cho, Unnan, Shimane 690-2404 Japan; Department of Biostatistics and Epidemiology, Graduate School of Medicine, Yokohama City University, 3-9 Fukuura, Kanazawa-ku, Yokohama, Kanagawa 236-0004 Japan; Department of Preventive Medicine and Public Health, Tokyo Medical University, 6-1-1, Shinjuku, Shinjuku-ku, Tokyo 160-8402 Japan; Department of Health and Social Behavior, School of Public Health, The University of Tokyo, 7-3-1 Hongo, Bunkyo-ku, Tokyo 113-0033 Japan; Department of Functioning Activation, National Centre for Geriatrics and Gerontology, 7-430 Morioka-machi, Obu, Aichi 474-8511 Japan; Department of Epidemiology, Harvard School of Public Health, 677 Huntington Avenue, Boston, MA 02115 USA; School of Public Health, The University of Sydney, Sydney, NSW 2006 Australia

**Keywords:** Walking, Muscle stretching exercises, Resistance training, Musculoskeletal diseases, Health communication

## Abstract

**Background:**

Promotion of physical activity (PA) is a key strategy to prevent non-communicable diseases. However, evidence on the effectiveness of community-wide interventions (CWIs) for promoting PA is limited.

**Purpose:**

To evaluate the effectiveness of a 3-year CWI for promoting PA in middle-aged and older adults compared with usual public health services. This study is an extension to an original 1-year investigation study.

**Design:**

Cluster randomized controlled trial with community as unit of randomization and individual as unit of analysis.

**Setting/participants:**

12 communities in Unnan, Japan were randomly allocated to the intervention (9) or the control (3). Additionally intervention communities were randomly allocated to aerobic activity promotion (Group A), flexibility and muscle-strengthening activities promotion (Group FM), or aerobic, flexibility, and muscle-strengthening activities promotion (Group AFM), each consisting of three communities. Randomly-sampled 4414 residents aged 40 to 79 years responded to the baseline survey (74 %), and were analyzed in 2013–2014.

**Intervention:**

A 3-year CWI based on social marketing, to promote PA from 2009 to 2012.

**Main outcome measures:**

The primary outcome was a change in regular aerobic, flexibility, and/or muscle-strengthening activities, defined by (1) engaging in 150 mins/week or more of walking, (2) engaging in daily flexibility activity, or (3) engaging 2 or more days/week in muscle-strengthening activities, evaluated at the individual level. Secondary outcomes were changes in specific types of PA and musculoskeletal pain. Outcomes were measured at baseline and at 1 and 3 years (2009, 2010, and 2012).

**Results:**

The CWI did not significantly increase the proportion of adults who reached recommended levels of aerobic, flexibility, and/or muscle-strengthening activities (adjusted change difference = 1.6 % [95 % CI: −3.5, 6.6]). In the subgroup analysis, compared to the controls, adults doing flexibility activity daily significantly increased in Group FM (6.3 % [95 % CI: 1.9, 10.7]). In Group A and AFM for PA outcomes and in all groups for pain outcomes, there was no significant change compared to controls.

**Conclusions:**

The CWI did not achieve significant increase in the proportion of adults who reached recommended PA levels. However, it might be effective in promoting flexibility activity in middle-aged and older Japanese.

**Trial registration:**

UMIN-CTR UMIN000002683.

**Electronic supplementary material:**

The online version of this article (doi:10.1186/s12966-015-0242-0) contains supplementary material, which is available to authorized users.

## Background

Physical activity (PA) reduces the risks of many non-communicable diseases [1–5]. However, physical inactivity is a common public health problem globally [6, 7].

Considering diverse factors affect PA at the individual, social, environmental, and policy level [8, 9], multilevel and intersectoral approaches are reasonable candidates for the whole-of-community PA promotion strategy to be examined [9, 10]. Recently, community-wide interventions (CWIs) have been implemented for promoting PA and examined for their effectiveness by research. Such CWI typically (1) involve many community sectors; (2) include highly visible, broad-based, multi-component strategies; and, (3) may also address other cardiovascular disease risk factors [11, 12]. However, evidence on the effectiveness of CWI for promoting PA, based on well-designed trials, is limited [13–23]. A Cochrane review first published in 2011 concluded that there was a lack of appropriate studies which could show whether this approach was beneficial [22]. An updated review published in 2015 [24] identified 4 high quality (low risk of bias) studies including our 1-year intervention study which was the first ever published study to examine the effectiveness of CWI in adults by a randomized controlled trial (RCT) [23]. However, overall, there was an absence of benefit in PA for CWIs in the included studies, as well as our study [24]. It is still unclear whether the reason of the absence of the evidence of benefit in PA for CWIs is based on the nature of the CWI itself or the dose, duration, or types of intervention components. Of note, the most frequent duration of the intervention in these studies was 1 year (median = 3 years) [24]. As it may take considerable time to achieve population-level improvement in PA, examining CWI in longer duration studies is important.

In terms of types of PA targeted by CWIs, most previous studies focused on only aerobic activity (e.g., walking) [12–21]. However, flexibility and muscle-strengthening activities are recommended for older adults and people with musculoskeletal disorders, as well as aerobic activity [25–29]. Musculoskeletal disorders are a major burden on both individuals and societies [30]. In addition, as arthritis is a potential barrier to PA, mainly aerobic activity [31], identifying effective strategies to promote flexibility and musculoskeletal activities and prevent musculoskeletal disorders is important.

Therefore, this study extended the original 1-year investigation trial and aimed to evaluate the effectiveness of a 3-year CWI for promoting not only aerobic PA, but also flexibility and muscle-strengthening activities in middle-aged and older adults using a cluster RCT. The intention was to promote PA through a CWI delivered at the community level. To minimize contamination, the unit of randomization was the community. The hypothesis was that a 3-year CWI delivered at the community level would promote engagement in recommended levels of aerobic, flexibility, and/or muscle-strengthening activities in middle-aged and older adults evaluated at the individual level.

## Methods

This study reports on findings after 3 years of intervention in the COMMUNICATE (COMMUNIty-wide CAmpaign To promote Exercise) study. This was originally a 1-year cluster randomized controlled, superiority trial, stratified by population density, with imbalanced randomization (3 interventions; 1 control) [23], where intervention was continued for a further 2 years when no significant effect on population-level change in PA was seen after the first year. The study location was Unnan City (population 45364, area 553.7 km^2^), Shimane, Japan. Full details of the trial protocol can be found elsewhere and the original 1-year trial showed short-term effects on the awareness and knowledge of the residents [23]. This study was approved by the research ethics committee of the Physical Education and Medicine Research Center UNNAN (H21-10-13-1).

**Fig. 1:**
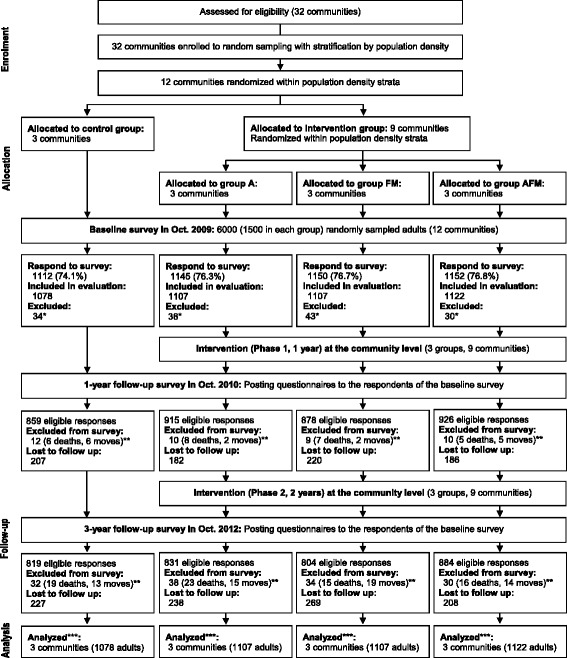
Flowchart of the study. *Note.* Group A, aerobic activity; Group FM, flexibility and muscle-strengthening activities; Group AFM, aerobic, flexibility, and muscle-strengthening activities. *Respondents who could not walk unaided. **Accumulated numbers of deaths and moves since baseline survey. ***Analyzed with missing data imputation

Figure 1 is a flow diagram of the trial. There are 32 communities within Unnan, with a median population and area of 1292 and 10.8 km^2^, respectively. The eligibility criterion for clusters was all communities in Unnan. Twelve communities (clusters) were randomly sampled, with stratification by blocking within population density category strata, and random allocation to three intervention clusters per control cluster (i.e., 9 interventions; 3 controls). Additionally each cluster in the intervention group was randomly allocated to aerobic activity promotion (Group A), flexibility and muscle-strengthening activities promotion (Group FM), or aerobic, flexibility, and muscle-strengthening activities promotion (Group AFM), each consisting of 3 clusters. This factorial designed division was for the purpose of subgroup analyses.

Random selection and allocation of the clusters were performed by 2 clerical staff members of Unnan City Hall, who were not involved in the remainder of the study. A staff member created a matched list of numbers (used later as selected community ID × assignment group ID) by using a computer-generated random numbers. Another prepared ID lists of 1) communities and 2) assignment group were not given to the staff member to conceal the actual allocation of each community. Then the other staff member used the created list of numbers and the ID lists to assign communities (clusters). Any other cluster selection process to minimize the risk of contamination (e.g., geographical distance between individual clusters) was not performed.

### Intervention

A CWI to promote PA for all middle-aged and older (40–79 years) residents living in the communities was conducted as an Unnan City Hall public health project at the cluster level within intervention groups for 3 years (November 2009 to October 2012). In Group A, mainly walking was promoted. In Group FM, stretching exercises and muscle-strengthening activities focused on key muscle groups for treating low back and knee pain were promoted [27, 32, 33]. In Group AFM, all of these walking, stretching, and muscle-strengthening activities were promoted.

The CWI adopted social marketing techniques [34]. The detailed strategy can be found elsewhere [23]. Briefly, the CWI adopted the following processes: 1) situational analysis; 2) market segmentation and targeting; 3) setting objectives; and 4) marketing strategy development. Based on the social marketing process, the common key message of “*Be active to cure your low back and knee pain*” (originally in Japanese) was selected for all intervention communities. Cooperative relationships were developed with local organizations including each community’s self-administered organization.

Throughout the intervention period, the CWI consisted of 3 components, namely:

1. Information delivery. Flyers, leaflets, community newsletters, posters, banners, and local audio broadcasts (sample posters available in Additional file 1).
2. Education delivery. Outreach health education program and mass- and individual encouragement by professionals during community events. Mass-encouragement included a motivating talk and demonstration of PA using a common procedure to ensure standardization of the intervention and individual encouragement included face-to-face promotion of PA while waiting for community health check-ups (sample informative thank-you cards for participants of health check-ups available in Additional file 2).
3. Support delivery. Development of social support, i.e., promoting encouragement by community leaders and lay health workers; material support, i.e., arranging for residents to obtain light-reflective material for walking safety, pedometers (Group A and AFM), and videotapes and DVDs on flexibility and muscle-strengthening activities (Group FM and AFM) at each relevant community center; and professional support, i.e., establishing a call center for questions about PA and requests for outreach programs.

Intervention was divided into 2 phases, phase 1: Nov 2009 - Oct 2010; phase 2: Nov 2010 - Oct 2012. Among 3 components of the CWI, information and education delivery were emphasized in Phase 1. This approach was considered to be successful in increasing awareness and knowledge of the residents [23]. The dose (awareness) of support delivery was lower than those of the other components in Phase 1 [23]. However, interviews with new uptakers of PA during Phase 1 suggested that such small amount of peer support enhancement was powerful enough to change some residents’ behavior, PA (unpublished data). Therefore in Phase 2, using social network in the community and word-of-mouth buzz marketing (i.e., support delivery) was emphasized to change residents’ actual behavior, PA. Key influencers (opinion leaders) for the network intervention [35] were identified by the combination of self-selection, staff-selected, positional approach (persons who occupied leadership positions such as a secretery of the community center), and, snowball method (index cases provided nominations of leaders who were in turn interviewed until no new leaders were identified) [36]. Key influencers were defined as either “salesman”, “maven”, or “connector”; in short, salesman is a person with the skills to persuade people, maven is one who accumulates knowledge and has information on lots of different types of PA, resources for PA, or health-related activities, and connector is a person with a knack of making friends and acquaintances and who knows lots of people [37]. These identified influencers were invited to become community partners, and then those who agreed were encouraged to coordinate campaign activities and distribute campaign information by word-of-mouth with materials (e.g., flyers) within their communities.

The CWI met the definition of a CWI as set out previously [22]. It was possible that residents travelled between the different communities for shopping, commuting, etc. To avoid contamination of the intervention, flyers and leaflets were delivered to the household directly, and the audio messages were only delivered to households in the intervention communities using the cable network (i.e., not radio or terrestrial TV). Educational activities were implemented only at community events in which all participants were residents from the relevant community. In the control communities, public health services were delivered by Unnan City Hall as usual.

### Population-based evaluation

The effectiveness was evaluated by population-based surveys designed as a representative cohort. As a baseline, self-administered questionnaires were mailed to random participants in October 2009. A computer-based resident registry system was used for random sampling. Sampling frame (eligible respondents) was all men and women aged 40 to 79 years living in the 12 communities. Those excluded were individuals in assisted living facilities, those who required long-term care, or those who could not complete the questionnaires themselves due to disability. Those unable to walk unaided were also excluded from the analyses. One- and 3-year follow-up questionnaires were mailed to the baseline respondents in October 2010 and 2012. Those who had died or moved were excluded from the follow-up surveys but were included in the analyses. There was no racial or gender bias in the selection of participants.

All respondents gave written informed consent to participate in these surveys at baseline. Both participants and data collectors were randomly sampled residents. Residents and the CWI collaborators (e.g., community self-administered organization staff) were blinded to (not informed about) the study design and hypothesis (i.e., the existence of the control group and cluster allocation) [38]. The implementing staff of the CWI (intervention providers), data analysts, and the Mayor, Vice-Mayor, supervisory employees, and public health nurses of Unnan City Hall were not blinded to the cluster allocation. The statistical analysis was undertaken unblinded.

### Measures

#### Primary outcome

The primary outcome was the change in engagement in regular PA (overall PA) evaluated at the individual level from baseline to 3-year follow-up. If respondents met any one of the following conditions, they were defined as “engaging in regular PA”: (1) engaging in 150 minutes/week or more of walking, (2) engaging in daily flexibility activity, or (3) engaging in muscle-strengthening activities two or more days/week. Questions about specific varieties of PA (e.g., walking) would theoretically demonstrate greater responsiveness to the intervention than comprehensive PA questionnaires [39], because the intervention promoted specific types of PA rather more generic PA. The thresholds were based on the PA recommendations from the American College of Sports Medicine and the American Heart Association [1, 25] and the U.S. Department of Health and Human Services [3]. Daily threshold for flexibility activity was chosen because flexibility activity had been recommended, preferably, on all days that aerobic or muscle-strengthening activity is performed [25].

Respondents were asked about the number of days per week and the mean number of minutes walked per day, for recreation and transport separately, to give the weekly total minutes of walking time. Frequency of flexibility activity was assessed categorically (daily, not daily but occasionally, not at all). The weekly number of days performed was asked for muscle-strengthening activity. An English translation of the original Japanese questionnaire is available in Additional file 3. Walking and flexibility questionnaires were adopted from the Shimane Study [40]. Both the test-retest reliability over 10 days and criterion-related validity with an accelerometer of the walking questionnaire were acceptable (Spearman’s r = 0.79 and 0.38, respectively) and has been described elsewhere [23, 40]. The test-retest reliability of the flexibility and muscle-strengthening activities were also acceptable (weighted kappa = 0.72 for flexibility and Spearman’s r = 0.75 for muscle-strengthening activity) [23].

#### Secondary outcomes

Musculoskeletal pain was evaluated to represent possible benefits or harm related to the CWI. The pain locations were shoulder, low back, and knee. Chronic musculoskeletal pain was defined as current pain lasting longer than 3 months within the past 12 months [41]. A visual analog scale (VAS) from 0 mm (no pain) to 100 mm (most intense pain) was used to assess pain intensity [42]. The test-retest reliability had moderate and acceptable values of Cohen’s kappa for chronic pain (0.68 for shoulder; 0.49 for low back; 0.72 for knee) and Spearman’s r for VAS scores (0.80 for shoulder; 0.70 for low back; 0.78 for knee) [23].

All outcomes were same as the pre-specified ones in the original 1-year trial. As covariates, body mass index (BMI) calculated from self-reported weight and height in kg/m^2^, self-rated health, years of education, employment status, engagement in farming, and chronic disease history were examined by the baseline questionnaire. Information on sex and age were also gathered from the resident registry system.

#### Implementation evaluation

For information delivery, the numbers of flyers, leaflets, posters, community newsletters, and banners distributed were recorded. The number of times and the duration of local audio broadcasts were also recorded. For education delivery, a case report form which included the number of attending participants was used. The quasi-population coverage rate for such educational activities was calculated as gross numbers of participants divided by the population aged 40–79 years in the relevant community. Finally, for support delivery, the implemented sub-components were recorded. The information on times and hours of visits to and conversations with residents, and number and characteristics of community partners (influencers) were collected only in Phase 2 according to the focus of the CWI.

### Statistical analysis

As for the original 1-year investigation, the planned sample size of 9 clusters and 4500 representative participants in the intervention, and 3 clusters and 1500 representative participants in the control were calculated on the assumption of a 50 % response rate (i.e., total 3000 analyzed participants) to detect an 8 % difference in change in regular PA between the intervention and control groups, taking into account the design effect by cluster randomization [23, 43]. Based on available data, the estimated rate of regular PA at baseline was 58 % with an estimated intracluster correlation coefficient of 0.00174. The chi-square test was used with imbalanced randomization (3:1), a two-sided 5 % significance level, and a power of 90 %.

#### Primary and secondary analyses

Multi-level analyses, taking into account the multiple measurements (3 time points: baseline, 1- and 3-year follow-ups), were performed. The change difference between 9 intervention and 3 control clusters was calculated for the primary outcome of regular PA (overall PA) from baseline to 3-year follow-up using a generalized linear mixed model (GLMM) with sex, age, BMI, self-rated health, years of education, employment, farming, chronic low back and knee pain, chronic disease history, community (cluster) where respondents lived, time effect, group allocation (intervention or control), and the interaction between time and group as fixed effects, and individuals as a random effect. Community (cluster) was included as fixed effect rather than the random effect because to do so, it was possible to adjust all community level confounders regardless of whether they were measured or not [44].

As secondary analyses, each intervention subgroup (Groups A, FM, and AFM) was compared with the control communities for primary outcome and changes in each of the different activities (e.g., walking) using the similar GLMM. Changes in chronic musculoskeletal pain prevalence and VAS pain scores for shoulder, low back, and knee were also analyzed by the similar GLMM with the further adjustment for baseline PA.

Analyses were performed on an intention-to-treat basis and included all baseline respondents who could walk unaided. Missing information, ranged from 1 % for self-rated health to 24 % for walking time, was imputed to minimize bias due to missing information and repeated four times, under the assumption of missing at random [45]. Each imputation was based on regression models including variables used in the analyses. The five imputed datasets were analyzed independently and combined for inference. Sensitivity analyses with 10 imputed datasets provided similar results. Thus, only the results from the primary 5 imputed datasets are presented here. Significance was set at p < .05. Analyses were carried out using SAS version 9.3 (SAS Institute Inc., Cary, NC).

## Results

### Implementation evaluation

Table 1 shows the dose of the implemented information, education, and support delivery. All of these three dimensions of the CWI were implemented in all intervention communities. According to the emphases of the CWI in each phase, most components of visual and audio information and educational activities were delivered more intensively in phase 1, compared with phase 2. For example, total quasi-population coverage rate of educational activities were 62 % in phase 1 and 24 % in phase 2, respectively. In contrast, visits to community centers and residents’ home by intervention staffs and conversations with community partners were intensively done in phase 2. On average, 10 times and 7 hours of visits and conversations were implemented in each community. Characteristics and example activities of the 114 community partners are found in Additional file 4. Of these, 92 (81 %) were female and 74 (65 %) aged 60–79 years. The numbers who were identified as community-level “salesmen”, “mavens”, and “connectors” were 24, 24, and 39, respectively (including overlaps due to their multiple talents and excluding 66 partners unsure for their talents). In most communities, material support was not implemented in phase 2 because they were considered to be less influential than the other components according to the interviews with a sample of residents after the 1-year follow up.

**Table 1:** Implementation of information, education, and support delivery in intervention subgroups: COMMUNICATE Study

	Group A		Group FM		Group AFM	
	Phase 1	Phase 2	Phase 1	Phase 2	Phase 1	Phase 2
Information delivery						
<Visual information>						
Flyers or leaflets (times distributed to all households)	4	1	3	1	3	1
Posters (numbers hung)	34	66	24	75	34	84
Community newsletters (times articles about CWI appeared)	2	1	1^a^	0^a^	2	0
Banners (numbers placed)	2	1	2	1^a^	2	2
<Audio information>						
Local audio broadcasts (times audio messages broadcasted)	12	10	12	10	12	10
Education delivery						
Times educational activities implemented	16	14	14	13	17	6
Numbers of participants, group total (A)	1200	589	1878	865	1532	313
(Population aged 40–79 years, group total (B))	2132		2743		2618	
Quasi-population coverage rate (A/B, %)	56	28	68	32	59	12
Support delivery						
<Social support>						
Promoting encouragement by community partners^b^	2/3	3/3	0/3	3/3	2/3	3/3
Times of visits to and conversations with residents	- ^c^	13	- ^c^	5	- ^c^	11
Total hours of visits to and conversations with residents	- ^c^	8.4	- ^c^	4.3	- ^c^	8.8
Number of community partners	- ^c^	18	- ^c^	11	- ^c^	8.3
<Material support >^d^						
Loan and selling of pedometers^b^	2/3	0/3	NA	NA	1/3	0/3
Distribution of light-reflective materials^b^	3/3	0/3	NA	NA	3/3	0/3
Loan of video tapes and DVDs on FM activities^b^	NA	NA	3/3	0/3	2/3	1/3
<Professional support>						
Establishment of a call center^e^	yes	yes	yes	yes	yes	yes

CWI = community-wide intervention; Group A = aerobic activity; Group FM = flexibility and muscle-strengthening activities; Group AFM = aerobic, flexibility, and muscle-strengthening activities; NA = not applicable. Phase 1: from November 2009 through October 2010; Phase 2: from November 2010 through October 2012. Numbers are average of 3 communities in each group unless noted otherwise

^a^There was no regular community newsletter published by the self-administered organization only in a community of Group FM. Apart from community newsletter, 14 blog articles about the local campaign were posted in the website of the community and 16 community-specific banners were created and placed in the community in Phase 2

^b^Numbers indicate the proportion of communities that implemented this strategy

^c^There is no available numbers for Phase 1 because the detail information was collected only in Phase 2 according to the focus of the campaign

^d^Implemented at community centers

^e^A call center was established in Unnan City Hall for all communities

The standard public health services in the 3 control communities included public-based medical health check-ups, health education classes about general lifestyle and disease prevention (14 classes and total 192 participants in phase 1, and 32 classes and 497 participants in phase 2), and *ad hoc* health counseling during the intervention period.

### Effectiveness evaluation

Data from a total of 4414 (73.6 %) respondents were analyzed in the intention-to-treat manner (Fig. 1). Baseline characteristics of the eligible respondents are presented in Table 2. No significant differences between the control and intervention communities were observed at baseline.

**Table 2:** Baseline characteristics of participants randomly selected from communities: COMMUNICATE Study

	Control	Intervention				*P* value^a^
		All	Group A	Group FM	Group AFM	
Cluster	3	9	3	3	3	
Residents, *n*	5235	14721	3700	5553	5468	0.64
Residents aged 40–79 years, *n*	2917	7493	2132	2743	2618	0.93
Population density, mean ± SD, /km^2^	131 ± 137	273 ± 371	433 ± 641	145 ± 46	240 ± 268	0.52
Evaluation participants (eligible response rate)	1078 (71.9)	3336 (74.1)	1107 (73.8)	1107 (73.8)	1122 (74.8)	0.85
Male	510 (47.3)	1540 (46.2)	522 (47.2)	517 (46.7)	501 (44.7)	0.51
Age, Mean ± SD, years	61.0 ± 10.6	60.7 ± 10.5	61.2 ± 10.7	60.1 ± 10.4	60.6 ± 10.5	0.29
40-59	471 (43.7)	1514 (45.4)	477 (43.1)	522 (47.2)	515 (45.9)	
60-79	607 (56.3)	1822 (54.6)	630 (56.9)	585 (52.8)	607 (54.1)	
Body mass index, Mean ± SD, kg/m^2^	22.5 ± 3.2	22.6 ± 3.1	22.8 ± 3.2	22.3 ± 2.9	22.6 ± 3.0	0.68
<18.5	83 (8.1)	226 (7.0)	62 (5.9)	88 (8.2)	76 (6.9)	
≥18.5 to <25	744 (72.2)	2352 (72.9)	770 (72.8)	804 (74.8)	778 (71.1)	
≥25	204 (19.8)	650 (20.1)	226 (21.4)	183 (17.0)	241 (22.0)	
Self-rated health						
Excellent/good	878 (81.9)	2722 (82.7)	885 (80.8)	902 (83.0)	935 (84.3)	0.20
Fair/poor	194 (18.1)	569 (17.3)	210 (19.2)	185 (17.0)	174 (15.7)	
Years of education, mean ± SD	11.5 ± 2.3	11.5 ± 2.4	11.5 ± 2.4	11.4 ± 2.3	11.5 ± 2.5	0.72
Employed	695 (69.6)	2101 (68.7)	665 (64.6)	711 (70.0)	725 (71.6)	0.58
Engagement in farming	552 (52.4)	1626 (49.7)	466 (42.7)	627 (58.2)	533 (48.4)	0.13
Chronic disease history^b^	659 (61.1)	2059 (61.7)	679 (61.3)	673 (60.8)	707 (63.0)	0.73

Group A = aerobic activity; Group FM = flexibility and muscle-strengthening activities; Group AFM = aerobic, flexibility, and muscle-strengthening activities. VAS = visual analog scale. Figures are numbers (percentages) before imputation of missing values. Sample sizes (denominators) vary due to missing values

^a^Comparison between control and intervention groups using the chi-square test for binary variables and Mann–Whitney *U*-test for categorical and continuous variables with non-normal distribution

^b^Having the following disease history: hypertension, hyperlipidemia, diabetes, hyperuricemia, cerebrovascular disease, heart disease, kidney and urologic diseases, liver disease, gastrointestinal disease, endocrine disease, cancer

**Fig. 2:**
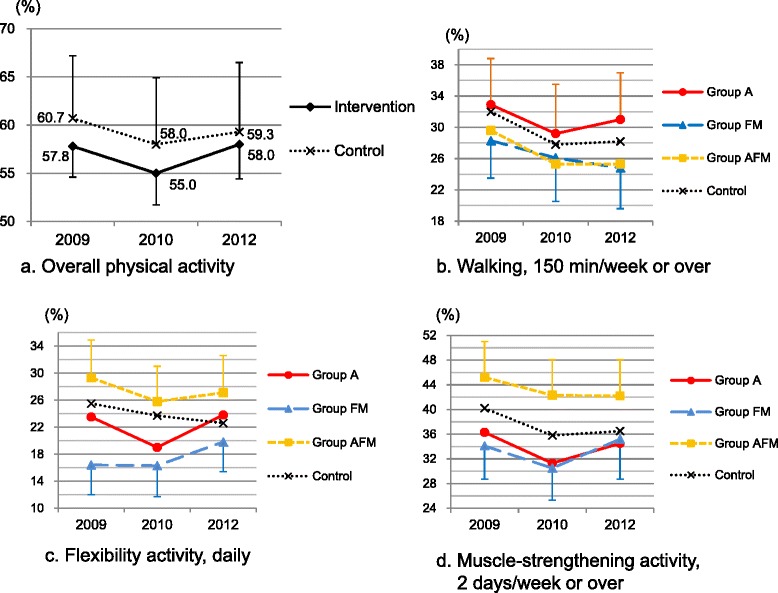
Adjusted prevalence of physical activity over the 3-year intervention period. Adjusted prevalence is shown for those met recommended level of overall physical activity (**a**), walked 150 min/week or over (**b**), engaged in flexibility activity daily (**c**), and engaged in muscle-strengthening activity for 2 days/week or over (**d**). In each community in the intervention subgroups, aerobic activity (Group A), flexibility and muscle-strengthening activities (Group FM), or all aerobic, flexibility, and muscle-strengthening activities (Group AFM) were promoted. The prevalence is adjusted for sex, age, body mass index, self-rated health, years of education, employment status, engagement in farming, chronic low back and knee pain, chronic disease history, and community (cluster) where the respondents lived; 95 % confidence intervals of specific physical activities (b, c, d) are presented only for groups with the lowest or highest prevalence over the period. Range of confidence-bar lengths for specific physical activities is 4.2-6.6

Table 3 shows unadjusted distribution of physical activity and pain outcomes at baseline and 3-year follow-up by using samples without imputation. Adjusted prevalence of PA in the intervention and control groups are presented in Fig. 2. Overall and each type of PA had negative trends (decreased) in the control communities, although most were not significant (Table 4). The primary analysis revealed that the CWI did not significantly increase the overall PA over the 3-year period (adjusted change difference of % those who met the recommendation between intervention and control = 1.6 % [95 % confidence interval (CI): −3.5, 6.6]). For changes in each of the different activities (e.g., walking), the intervention effect was not significant, although all of them had positive values of adjusted change difference (0.6 to 3.4). In the subgroup analysis, compared to the control communities, the proportion of adults doing flexibility activity daily significantly increased in Group FM (adjusted change difference: 6.3 % [1.9, 10.7]). Adjusted change of flexibility activity within Group FM was 3.4 % (95 % CI: 0.4, 6.5) and significant. Not significant but positive and the largest effect sizes were also found for walking in Group A and for muscle-strengthening activity in Group FM, respectively. In Group AFM, there was no significant change compared with the control. In addition, there was no significant change difference in pain outcomes, neither prevalence or intensity, between intervention and control groups (Additional files 5 and 6).

**Table 3:** Unadjusted distribution of physical activity and pain outcomes at baseline and 3-year follow-up: COMMUNICATE Study

	Control	Intervention				*P* value^a^
		All	Group A	Group FM	Group AFM	
Overall regular physical activity^b^, *n*						
At baseline	573 (64.5)	1745 (63.0)	614 (66.6)	526 (58.3)	605 (64.0)	0.40
At 3 year	439 (61.8)	1380 (61.9)	481 (63.4)	416 (60.1)	483 (62.0)	
Total walking time, mins/week						
Median (IQR) at baseline	60 (0–210)	60 (0–200)	80 (0–210)	60 (0–180)	60 (0–200)	0.53
Median (IQR) at 3 year	60 (0–200)	60 (0–210)	90 (0–235)	40 (0–180)	60 (0–185)	
≥150, *n* at baseline	311 (37.7)	914 (36.4)	319 (38.1)	282 (34.1)	313 (37.0)	
≥150, *n* at 3 year	222 (33.8)	700 (34.6)	260 (37.9)	207 (32.5)	233 (33.4)	
Flexibility activity daily, *n*						
At baseline	253 (24.4)	772 (23.8)	276 (25.9)	214 (19.8)	282 (25.8)	0.45
At 3 year	175 (22.0)	603 (24.6)	213 (26.1)	183 (23.6)	207 (24.0)	
Muscle-strengthening activity, days/week						
Median (IQR) at baseline	0 (0–3)	0 (0–3)	1 (0–3)	0 (0–3)	0 (0–3)	0.99
Median (IQR) at 3 year	0 (0–3)	0 (0–3)	0 (0–4)	0 (0–3)	0 (0–3)	
≥2, *n* at baseline	348 (38.0)	1080 (37.7)	390 (40.9)	310 (33.1)	380 (39.2)	
≥2, *n* at 3 year	257 (32.8)	862 (35.8)	306 (38.3)	250 (32.8)	306 (36.1)	
Median (IQR) VAS pain score						
Shoulder at baseline	20 (0–48)	22 (0–48)	22 (0–49)	22 (0–48)	20 (0–48)	0.35
Shoulder at 3 year	11 (0–41)	14 (0–43)	13 (0–41)	17 (0–47)	12 (0–43)	
Low back at baseline	5 (0–32)	8 (0–36)	8 (0–36)	9 (0–37)	7 (0–32)	0.11
Low back at 3 year	4 (0–27)	4 (0–29)	4 (0–28)	5 (0–30)	3 (0–29)	
Knee at baseline	0 (0–7)	0 (0–13)	0 (0–15)	0 (0–11)	0 (0–12)	0.067
Knee at 3 year	0 (0–9)	0 (0–13)	0 (0–12)	0 (0–14)	0 (0–12)	
Chronic musculoskeletal pain						
Shoulder at baseline	158 (15.3)	554 (17.4)	176 (16.6)	203 (19.4)	175 (16.3)	0.11
Shoulder at 3 year	157 (19.7)	436 (17.8)	145 (18.0)	156 (20.0)	135 (15.7)	
Low back at baseline	133 (13.0)	441 (14.0)	145 (13.9)	150 (14.4)	146 (13.8)	0.43
Low back at 3 year	108 (13.6)	369 (15.0)	114 (14.0)	124 (15.8)	131 (15.1)	
Knee at baseline	95 (9.1)	360 (11.2)	115 (10.8)	122 (11.4)	123 (11.4)	0.062
Knee at 3 year	87 (11.0)	334 (13.6)	122 (15.0)	99 (12.6)	113 (13.3)	

Group A = aerobic activity; Group FM = flexibility and muscle-strengthening activities; Group AFM = aerobic, flexibility, and muscle-strengthening activities; IQR = interquartile range; VAS = visual analog scale. Figures are numbers (percentages) before imputation of missing values unless stated otherwise. Sample sizes (denominators) vary due to missing values

^a^Baseline comparison between control and intervention groups using the chi-square test for binary variables and Mann–Whitney *U*-test for categorical and continuous variables with non-normal distribution

^b^Engagement in regular aerobic, flexibility, and/or muscle-strengthening activities. If respondents met any one of the following three conditions, the respondents were defined as “engaging in regular physical activity”: (1) engaging in 150 mins/week or more of walking, (2) engaging in daily flexibility activity, or (3) engaging 2 or more days/week in muscle-strengthening activities

**Table 4:** Changes in physical activity from baseline to 3-year follow-up: COMMUNICATE Study

	ICC^a^	Control (*n* = 1078)	Intervention (n = 3336)	Subgroups^b^		
				Group A (*n* = 1107)	Group FM (n = 1107)	Group AFM (n = 1122)
Overall regular physical activity, % of those meet recommendation^c^	0					
Adjusted change within group		−1.4 (−5.3, 2.5)	0.1 (−2.3, 2.6)	−0.5 (−4.4, 3.4)	2.7 (−1.0, 6.4)	−1.7 (−5.7, 2.3)
Adjusted change difference		(ref)	1.6 (−3.5, 6.6)	0.9 (−5.2, 7.0)	4.1 (−1.4, 9.6)	−0.3 (−6.2, 5.6)
Specific physical activity						
Walking, % of those walking 150 min/w or over	0.0019					
Adjusted change within group		−3.9 (−7.8, 0.1)	−3.2 (−5.5, −1.0)**	−2.0 (−5.5, 1.6)	−3.5 (−7.0, 0.1)	−4.3 (−9.9, 1.3)
Adjusted change difference		(ref)	0.6 (−4.2, 5.4)	1.9 (−3.4, 7.2)	0.4 (−4.7, 5.4)	−0.5 (−8.1, 7.2)
Flexibility activity, % of those engaging in daily	0.00033					
Adjusted change within group		−2.9 (−5.9, 0.1)	0.5 (−1.2, 2.2)	0.3 (−2.9, 3.6)	3.4 (0.4, 6.5)*	−2.2 (−5.8, 1.4)
Adjusted change difference		(ref)	3.4 (−0.1, 6.9)	3.2 (−1.1, 7.5)	6.3 (1.9, 10.7)**	0.7 (−4.0, 5.4)
Muscle-strengthening activity, % of those engaging 2 days/w or over	0.0025					
Adjusted change within group		−3.7 (−7.3, −0.1)*	−1.2 (−3.5, 1.1)	−1.8 (−6.1, 2.5)	1.2 (−3.2, 5.6)	−3.0 (−6.4, 0.4)
Adjusted change difference		(ref)	2.5 (−1.6, 6.6)	1.9 (−3.2, 7.0)	4.9 (−0.7, 10.5)	0.7 (−4.3, 5.7)

Group A = aerobic activity; Group FM = flexibility and muscle-strengthening activities; Group AFM = aerobic, flexibility, and muscle-strengthening activities. Estimates are adjusted for sex, age, body mass index, self-rated health, years of education, employment status, engagement in farming, chronic low back and knee pain, chronic disease history, and community (cluster) where respondents lived. Numbers are presented with their 95 % confidence intervals in parentheses. An adjusted change difference greater than zero signifies that the intervention had a positive effect (favorable for physical activity) compared with the control group. *P < 0.05; **P < 0.01

^a^Intracluster correlation coefficient (ICC) of each outcome variable at 3-year follow-up was calculated by using samples without imputation as follows: ICC = (BMS - WMS)/(BMS + [K - 1] WMS), where BMS is the between-cluster mean square, WMS is the within-cluster mean square, and K is the average number of respondents per cluster. ICC is displayed as zero if the estimated value is smaller than zero

^b^All subgroups were analyzed simultaneously

^c^Engagement in regular aerobic, flexibility, and/or muscle-strengthening activities. If respondents met any one of three following conditions, the respondents are defined as “engaging in regular physical activity”: (1) engaging in 150 mins/week or more of walking, (2) engaging in daily flexibility activity, or (3) engaging 2 or more days/week in muscle-strengthening activities

## Discussion

The 3-year CWI did not increase overall PA as the primary outcome. However, the results also suggested that the CWI might be effective in promoting flexibility activity. Not significant but suggestive increases in walking and muscle-strengthening activities were observed in the communities where such activities were also promoted. To our knowledge, the original 1-year investigation of the COMMUNICATE study [23] is the first published study that examined the effectiveness of a CWI for promoting PA in middle-aged and older adults by using a cluster randomized design [24], and this study adds knowledge on 3-year evaluation results.

It is not known how long a CWI should be conducted to increase PA at the community level. The original 1-year investigation showed short-term effects on awareness and knowledge [23], and the current 3-year evaluation suggests trends towards actual behavior change. Baker et al. proposed that awareness and knowledge change as short-term impacts, and changes in belief, intention, and PA level are medium-term outcomes of CWIs [22, 24]. The most frequent duration of interventions in the 33 studies in the review was 1 year and the median duration was 3 years with a range of 1–7 years [24]. The results of this study suggest that 3 years or longer duration might be needed for CWIs to achieve population-level behavior (PA) change.

In addition to the duration, the dose of intervention required for population-level increase of PA is unknown. This study presents information on the implementation process. In Group FM, where significant increase in the proportion reporting flexibility activity was observed, quasi-population coverage rate of the educational activities was relatively high, compared with the other groups. Although an effort to deliver intervention components equally at the same level of dose across all communities was an essential part of this community intervention trial, implementation dose typically can vary by communities in this kind of trials and the difference in the dose of educational activities among subgroups might be one of the factors which led to the differences in behavior changes of the residents. By contrast, doses of some social support component were relatively higher in Group A and AFM than Group FM. This component was emphasized later time period (phase 2) and was an indirect approach to reach residents. Therefore, the expected effect of this component on residents’ PA might need more time to be observed.

When viewed from another perspective, the results of this study also demonstrate the difficulty in whole community level PA improvement by the implemented approaches. The implemented CWI included multi-dimensional approaches but more comprehensive approach including policy and built environmental change strategies such as creation or enhancement of public transport systems and exercise infrastructure might be necessary to achieve population-level PA increase [12, 22].

In this study, no result supported the “all-in-one” intervention (i.e., Group AFM) was more effective than the targeted ones (Group A and FM). In Group AFM where all three types of PA were promoted, the amount of information delivered was greater than that for Group A and FM, thus the burden on the residents might also be greater. If the CWI could succeed in motivating older people to perform all types of PAs, then the achieved health benefit would be the greatest. [1, 25] In order to disseminate the current PA recommendations, which include multiple types of PA, to the lay public, interventions should focus the types of PA to be promoted and a phased strategy (e.g., flexibility activity in the first phase and aerobic activity in the second phase) might work better to include all types of PA.

No significant change difference was observed in pain outcomes. As the logic model hypothesized that pain improvement occurs after PA increase [23], this is not unexpected. It can also be considered that no harm was observed. Both too little and too much PA have been suggested as potential risks for musculoskeletal pain [46, 47]. Monitoring musculoskeletal disorders is important to assess the potential harm of PA intervention.

This study has several strengths. First, a cluster RCT with the whole community level measurement is considered as the optimal design to develop practice-based evidence [48], and it is a clear strength of this study. Second, the prospective cohort design enabled individual level analysis and had more statistical power compared with multiple cross-sectional sampling. A potential disadvantage of this design is the risk of attrition bias. However, the high response rate with the adoption of the established methods to increase response rate [23, 49] provided less risk for biased results. As noted above, detection of potential harm using a pain questionnaire, which is not usually considered in PA interventions, is also strength of this study. Finally, this study examined all aspects of the *RE-AIM* framework (i.e., *Reach*, *Effectiveness*, *Adoption*, and *Implementation*) [50] except for the *Maintenance* aspect as it is an ongoing project. They are useful to evaluate the public health impact of the CWI. Collaborations with community organizations and utilization of existing resources realized high (100 %) adoption and implementation rate of the CWI components, and its good implementation adherence represent high applicability of the CWI to other locations. The standardized protocol and training of core team staff members [23] might also contribute to the quality control of the CWI between communities.

However, there also are limitations. First, a self-administered questionnaire might be subject to recall bias. In smaller-scale trials, objective measures (e.g., accelerometers) can be used as outcome measurement [51]. However, in broader-reach trials, objective measures are often prohibitively expensive, burdensome to participants and logistically difficult. Therefore, in broad-reach trials, self-report measures frequently must be relied on and brief self-report measures have been suggested as useful for their comparability of population PA estimates and have low respondent burden [52]. In addition, little is known to date about objective methods to assess flexibility and muscle-strengthening activities in population-wide studies. Using questionnaires with acceptable reliability to measure these activities is a strength of this study.

Second, minimizing the potential contamination of social network dissemination is more difficult than for information and education delivery. Although we assumed older adults mostly interacted within their communities, it is possible that contamination between communities might have occurred especially in the late phase.

Finally, the number of clusters allocated to each study arm was relatively small, although the cluster size was based on the sample size calculation. Partly due to its small number of clusters, significant differences in baseline proportions of flexibility and muscle-strengthening activities between groups were observed after adjustment (max 12.9 % and 11.1 % differences, respectively, see Fig. 2). It is possible that the observed change difference of flexibility activity between Group FM and the control might be caused by regression to the mean. However, trends of changes in PA differed by types of PA and the indicated largest effect sizes in the relevant communities, where such activities were promoted, suggested that the intervention had some effect on the residents’ PA. Future study with more clusters and random allocation of clusters with stratification by baseline PA prevalence would provide less biased results.

## Conclusions

The 3-year CWI did not achieve a significant increase in the proportion of adults who reached recommended PA levels, similar to what we observed in the shorter (1-year) intervention. However, it might be effective in promoting flexibility activity in middle-aged and older Japanese. The current study, with a randomized design and output evaluation provides valuable information about the difficulties in demonstrating effectiveness and challenges to implementation of a CWI to attain population-level increase of PA.

## Additional files

Additional file 1:
**Poster.** Sample materials (posters) of the community-wide intervention: COMMUNICATE Study (Phase 2, 2010–2012). Additional file 2:
**Thank-you cards.** Sample materials (informative thank-you cards for participants of health check-ups) of the community-wide intervention: COMMUNICATE Study (Phase 2, 2010–2012). Additional file 3:
**Physical activity questionnaire (English version).** An English translation of the original Japanese questionnaire. Additional file 4:
**Community partners.** Descriptive characteristics and example activities of community partners (N = 114). Additional file 5:
**Table of pain outcomes.** Changes in musculoskeletal pain from baseline to 3-year follow-up: COMMUNICATE Study. Additional file 6:
**Figure of pain outcomes.** Adjusted prevalence of chronic musculoskeletal pain over the 3-year intervention period.

## Abbreviations

PA: Physical activity
CWI: Community-wide intervention
RCT: Randomized controlled trial
Group A: Aerobic activity group
Group FM: Flexibility and muscle-strengthening activities group
Group AFM: Aerobic, flexibility, and muscle-strengthening activities group
VAS: Visual analog scale
BMI: Body mass index
GLMM: Generalized linear mixed model
CI: Confidence interval
