# Supplementary material for: Community-wide promotion of physical activity in middle-aged and older Japanese: a 3-year evaluation of a cluster randomized trial
Source: Int J Behav Nutr Phys Act. 2015 Jun 23;12:82. doi: 10.1186/s12966-015-0242-0 (PMC4484628; doi:10.1186/s12966-015-0242-0)
Supplement: Additional file 1: — Poster. Sample materials (posters) of the community-wide intervention: COMMUNICATE Study (Phase 2, 2010–2012). [file 12966_2015_242_MOESM1_ESM.pdf]

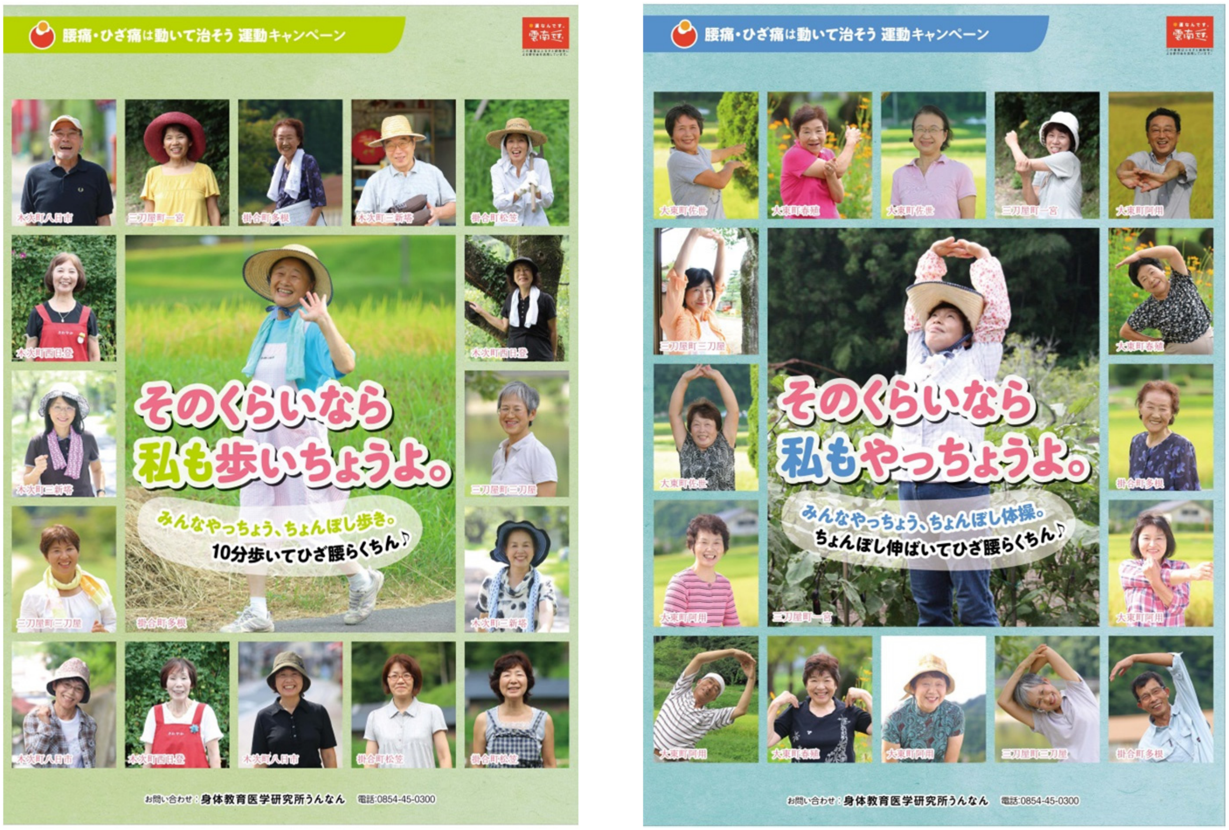

Additional file 1: Figure. Sample materials (posters) of the community-wide intervention: COMMUNICATE Study (Phase 2, 2010–2012).

Left: Walking for Group A and AFM. Main message: "If such a short distance is fine, I'm a regular walker, too." Everybody walks for a short distance. Walk for just 10 minutes for better lower back and knees.

Right: Flexibility activity for Group FM and AFM. Main message: "If such a small effort is fine, I'm an exerciser, too." Everybody does short-time taisou [flexibility and muscle-strengthening activities]. You flex your body, your low back and knees get better.

Both, at top: "Be active to cure your low back and knee pain."

(All in the Unnan dialect of Japanese)
