# Supplementary material for: Community-wide promotion of physical activity in middle-aged and older Japanese: a 3-year evaluation of a cluster randomized trial
Source: Int J Behav Nutr Phys Act. 2015 Jun 23;12:82. doi: 10.1186/s12966-015-0242-0 (PMC4484628; doi:10.1186/s12966-015-0242-0)
Supplement: Additional file 2: — Thank-you cards. Sample materials (informative thank-you cards for participants of health check-ups) of the community-wide intervention: COMMUNICATE Study (Phase 2, 2010–2012). [file 12966_2015_242_MOESM2_ESM.pdf]

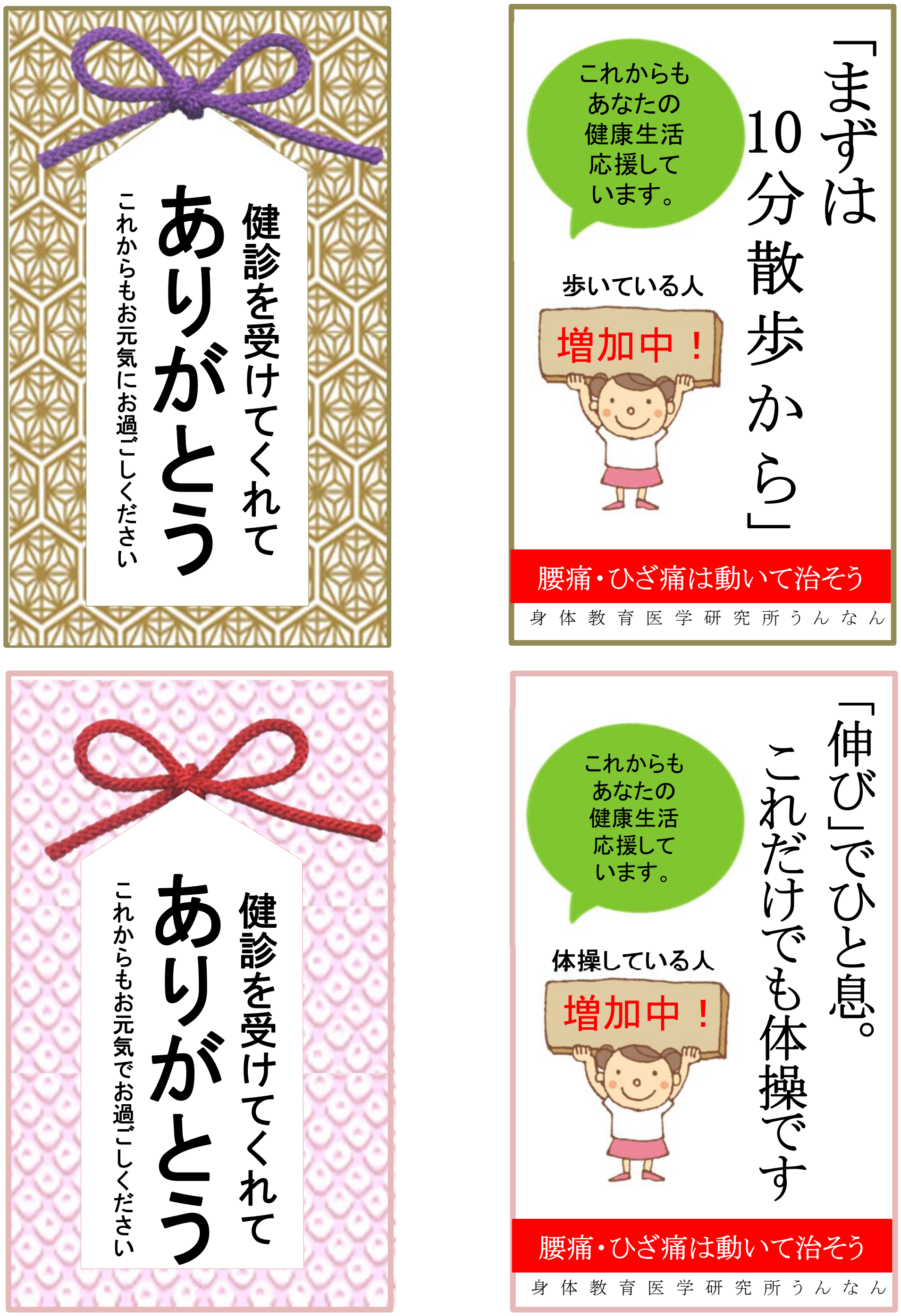

(A, front) (A, back)

(B, front) (B, back)

Additional file 2: Figure. Sample materials (informative thank-you cards for health check-up participants) for the community-wide intervention: COMMUNICATE Study (phase 2, 2010–2012).
A: Walking for Group A and AFM. Main message: Thank you for taking a health check-up. (front) Start with walking for 10 minutes. We are supporting your healthy life. The number of walkers is on the rise! (back)
B: Flexibility activity for Group FM and AFM. Main message: Thank you for taking a health check-up. (front) A break with a stretch, this is also a kind of taisou [flexibility and muscle-strengthening activities]. We are supporting your healthy life. The number of people participating in taisou is on the rise! (back)
At the bottom on the reverse side of both: “Be active to cure your low back and knee pain.”
(All in Japanese)
