# Supplementary material for: Community-wide promotion of physical activity in middle-aged and older Japanese: a 3-year evaluation of a cluster randomized trial
Source: Int J Behav Nutr Phys Act. 2015 Jun 23;12:82. doi: 10.1186/s12966-015-0242-0 (PMC4484628; doi:10.1186/s12966-015-0242-0)
Supplement: Additional file 3: — Physical activity questionnaire (English version). An English translation of the original Japanese questionnaire. [file 12966_2015_242_MOESM3_ESM.pdf]

Additional file 3. Physical activity questionnaire (English version)

[Walking]

9. How often/long do you walk for recreation or exercise (including dog walking)?

*Write ”0”, if you do not. ☐ days/week ☐ minutes/day

10. Do you usually walk for transport for more than 10 minutes at a time (e.g., for commuting, going to hospitals, shops, or your rice/crop fields, going on an errand to a community center or in the neighborhood)?
(Exclude walking while shopping and walking for recreation answered in the above question 1.)

*Write ”0”, if you do not. ☐ days/week ☐ minutes/day

[Flexibility activity]

11. Do you engage in flexibility activity (i.e., stretching exercises) or any activities to stretch or flex your body?

1. Daily (at least once a day)	2. Not daily but occasionally	3. not at all

[Muscle-strengthening activity]

12. Do you usually do activities to maintain and/or improve muscles and/or muscle strength? (e.g., sit-ups, squats, knee extensions)

*Write ”0”, if you do not. ☐ days/week

This is an English translation of the original Japanese questionnaire. The original Japanese version is available as an additional file 1 (protocol, Appendix 12) of the 1-year investigation paper (Kamada M et al., *Int J Behav Nutr Phys Act* 2013, 10:44.) at http://www.ijbnpa.org/content/supplementary/1479-5868-10-44-s1.pdf.
