# Supplementary material for: Community-wide promotion of physical activity in middle-aged and older Japanese: a 3-year evaluation of a cluster randomized trial
Source: Int J Behav Nutr Phys Act. 2015 Jun 23;12:82. doi: 10.1186/s12966-015-0242-0 (PMC4484628; doi:10.1186/s12966-015-0242-0)
Supplement: Additional file 4: — Community partners. Descriptive characteristics and example activities of community partners (N = 114). [file 12966_2015_242_MOESM4_ESM.pdf]

Additional file 4: Table. Descriptive characteristics and example activities of community partners (N=114)

	PD category	Sex		Age, years			Influencer category [a]				Community partners and example activities (relevant influencer category)
		Male	Female	40–59	60–79	80+	S	M	C	Un	
Group A											
COM 1	High	1	15	1	13	2	4	5	7	6	2 secretaries and 3 staff members of the community center organized campaign activities, sent information about events to all households in the community in the form of a newsletter, and personally invited residents to participate in the events. (S, M, C)<br>8 executive members of the community's self-administered organization established a monthly walking group and carried a campaign banner and wore hand-made campaign sashes as they walked. (C)<br>3 senior exercise volunteers introduced their tips on active living to community members at community meetings and health education classes and invited their neighbors to join exercise groups. (S)
COM 2	Middle	2	18	0	20	0	2	2	4	16	1 secretary and 2 staff members of the community center sent information about campaign activities (e.g., health education classes) to all households in the community in the form of a newsletter. (C)<br>7 members of a monthly health-activity circle distributed information about the campaign activities to their neighbors. (S, M, C)<br>10 participants in a health education class agreed to publicize the campaign by word of mouth and provided information to their neighbors in the form of informative flyers. (C)
COM 3	Low	1	18	13	5	1	4	6	6	13	15 members of a women's group who were highly interested in health-related information distributed campaign-related information (e.g., tips on walking for health) among their neighbors. (S, M, C)<br>3 family members who managed a grocery store, which also functioned as a post office, and a mobile retail truck provided information about walking as a health activity to their customers and neighbors. (S, M, C)<br>1 secretary of the community center set up walking events and involved various community groups in the campaign. (S, C)
Group FM											
COM 4	High	0	14	0	11	3	1	1	1	13	1 executive member of the community's senior women's group who was highly interested in health-related information distributed campaign-related information among her neighbors. (S, M, C)<br>13 members of the community's senior women's group agreed to publicize the campaign by word of mouth and provided information to their neighbors in the form of informative flyers. (C)
COM 5	Middle	0	1	1	0	0	1	1	1	0	1 secretary of the community center who was formerly a preschool principal and was highly interested in health-related information distributed campaign-related information throughout the community in the form of informative flyers and posters; she also demonstrated FM activities at various events of the senior citizens' club. (S, M, C)
COM 6	Low	16	3	11	8	0	2	2	3	16	1 secretary and 1 staff member of the community center developed and promoted the community's original FM exercises with the assistance of intervention staff and received public funding from city hall. (S, M, C)<br>16 voluntary members of the health department of the community's self-administered organization learned the original FM exercises and demonstrated them at various meetings and events on an organized basis.<br>1 senior exercise volunteer learned the original FM exercises and demonstrated them in exercise groups. (C)
Group AFM											
COM 7	High	0	13	2	11	0	5	2	9	0	8 participants of an exercise class agreed to publicize the campaign by word of mouth and provided information to their neighbors in the form of informative flyers. (S, C)<br>2 residents participated in an exercise class and invited many of their friends and neighbors to join. (S)<br>3 senior exercise volunteers posed for photographs used in flyers and posters, supplied campaign-related information to their neighbors, and invited them to participate in exercise classes. (M, C)
COM 8	Middle	1	4	3	2	0	4	3	3	0	1 secretary and 1 staff member of the community center distributed campaign-related information to community residents at various events. (S, C)<br>3 senior exercise volunteers posed for photographs used in flyers and posters, provided campaign-related information to their neighbors, and invited them to participate in exercise classes. (S, M)
COM 9	Low	1	6	2[b]	4	1	1	2	5	2	5 residents in the community helped with the distribution of posters and persuaded their neighbors to hang them up. (S, C)<br>1 staff member of the regional social welfare council coordinated the campaign activities and distributed and hung up the posters. (C)<br>1 staff member of the community center learned FM exercises and demonstrated them at 7 monthly chat salons for older adults. (S, C)
Total		22	92	33	74	7	24	24	39	66	

Notes. (Group) A, aerobic activity; AFM, aerobic, flexibility, and muscle-strengthening activities; COM #, community ID; FM, flexibility and muscle-strengthening activities; PD, population density. Numbers indicate the number of community partners for each category.

[a] Community partners were categorized based on their characteristics as influencers by the intervention staff. Some partners appear in multiple categories owing to their various abilities. S, salesman; M, maven; C, connector; Un, unsure due to insufficient information in the recruiting process.

[b] Includes one male in his 20s.
