# Supplementary material for: Community-wide promotion of physical activity in middle-aged and older Japanese: a 3-year evaluation of a cluster randomized trial
Source: Int J Behav Nutr Phys Act. 2015 Jun 23;12:82. doi: 10.1186/s12966-015-0242-0 (PMC4484628; doi:10.1186/s12966-015-0242-0)
Supplement: Additional file 5: — Table of pain outcomes. Changes in musculoskeletal pain from baseline to 3-year follow-up: COMMUNICATE Study. [file 12966_2015_242_MOESM5_ESM.pdf]

Additional file 5: Table. Changes in musculoskeletal pain from baseline to 3-year follow-up—COMMUNICATE Study

	ICC [a]	Control (n=1078)	Intervention (n=3336)	Subgroups [b]		
				Group A (n=1107)	Group FM (n=1107)	Group AFM (n=1122)
Chronic shoulder pain, %	0.00081					
Adjusted change within group		**3.5 (0.7, 6.4)***	0.5 (–1.3, 2.3)	1.4 (–1.7, 4.5)	–0.1 (–2.8, 2.6)	0.3 (–2.5, 3.0)
Adjusted change difference		(ref)	–3.0 (–6.6, 0.5)	–2.1 (–6.5, 2.3)	–3.7 (–7.7, 0.4)	–3.3 (–7.4, 0.9)
Chronic low back pain, %	0.00021					
Adjusted change within group		0.1 (–2.5, 2.7)	0.5 (–1.1, 2.2)	0.4 (–2.6, 3.3)	0.3 (–3.0, 3.6)	0.8 (–1.7, 3.3)
Adjusted change difference		(ref)	0.4 (–3.0, 3.8)	0.3 (–3.9, 4.5)	0.2 (–4.4, 4.8)	0.7 (–3.0, 4.4)
Chronic knee pain, %	<0.0001					
Adjusted change within group		1.8 (–0.6, 4.2)	1.4 (–0.1, 2.9)	2.4 (–0.2, 5.0)	0.5 (–1.9, 2.8)	1.4 (–1.0, 3.8)
Adjusted change difference		(ref)	–0.4 (–3.3, 2.6)	0.6 (–3.0, 4.2)	–1.3 (–4.9, 2.2)	–0.4 (–3.8, 3.1)
VAS shoulder pain score	0.0010					
Adjusted change within group		**−3.4 (−5.4, −1.5)****	**−3.2 (−4.3, −2.1)****	**−4.6 (−6.4, −2.7)****	−1.7 (−3.7, 0.2)	**−3.2 (−5.2, −1.2)****
Adjusted change difference		(ref)	0.3 (−2.1, 2.6)	−1.1 (−4.0, 1.7)	1.7 (−0.9, 4.3)	0.2 (−2.7, 3.1)
VAS low back pain score	0					
Adjusted change within group		−1.6 (−3.4, 0.3)	**−2.0 (−3.1, −1.0)****	**−2.5 (−4.2, −0.8)****	**−2.3 (−4.4, −0.2)***	−1.4 (−3.0, 0.2)
Adjusted change difference		(ref)	−0.5 (−2.8, 1.8)	−0.9 (−3.4, 1.6)	−0.8 (−4.0, 2.4)	0.2 (−2.2, 2.5)
VAS knee pain score	0					
Adjusted change within group		0.8 (−0.7, 2.3)	−0.0 (−0.9, 0.9)	−0.7 (−2.1, 0.8)	1.2 (−0.2, 2.5)	−0.6 (−2.2, 1.0)
Adjusted change difference		(ref)	−0.8 (−2.7, 1.0)	−1.5 (−3.6, 0.7)	0.4 (−1.7, 2.5)	−1.4 (−3.8, 0.9)

Notes. Group A, aerobic activity; Group FM, flexibility and muscle-strengthening activities; Group AFM, aerobic, flexibility, and muscle-strengthening activities; VAS, visual analog scale. Estimates are adjusted for sex, age, body mass index, self-rated health, years of education, employment status, engagement in farming, baseline overall physical activity, chronic disease history, and community (cluster) where respondents lived. Numbers are presented with their 95% confidence intervals in parentheses. An adjusted change difference greater than zero signifies that the intervention had a negative effect (increased pain prevalence or intensity score) compared with the control group. *P<0.05; **P<0.01

[a] Intracluster correlation coefficient (ICC) of each outcome variable at 3-year follow-up was calculated by using samples without imputation as follows: ICC = (BMS – WMS)/(BMS + [K – 1] WMS), where BMS is the between-cluster mean square, WMS is the within-cluster mean square, and K is the average number of respondents per cluster. ICC is displayed as zero if the estimated value is smaller than zero.

[b] All subgroups were analyzed simultaneously.
