# Supplementary material for: Community-wide promotion of physical activity in middle-aged and older Japanese: a 3-year evaluation of a cluster randomized trial
Source: Int J Behav Nutr Phys Act. 2015 Jun 23;12:82. doi: 10.1186/s12966-015-0242-0 (PMC4484628; doi:10.1186/s12966-015-0242-0)
Supplement: Additional file 6: — Figure of pain outcomes. Adjusted prevalence of chronic musculoskeletal pain over the 3-year intervention period. [file 12966_2015_242_MOESM6_ESM.pdf]

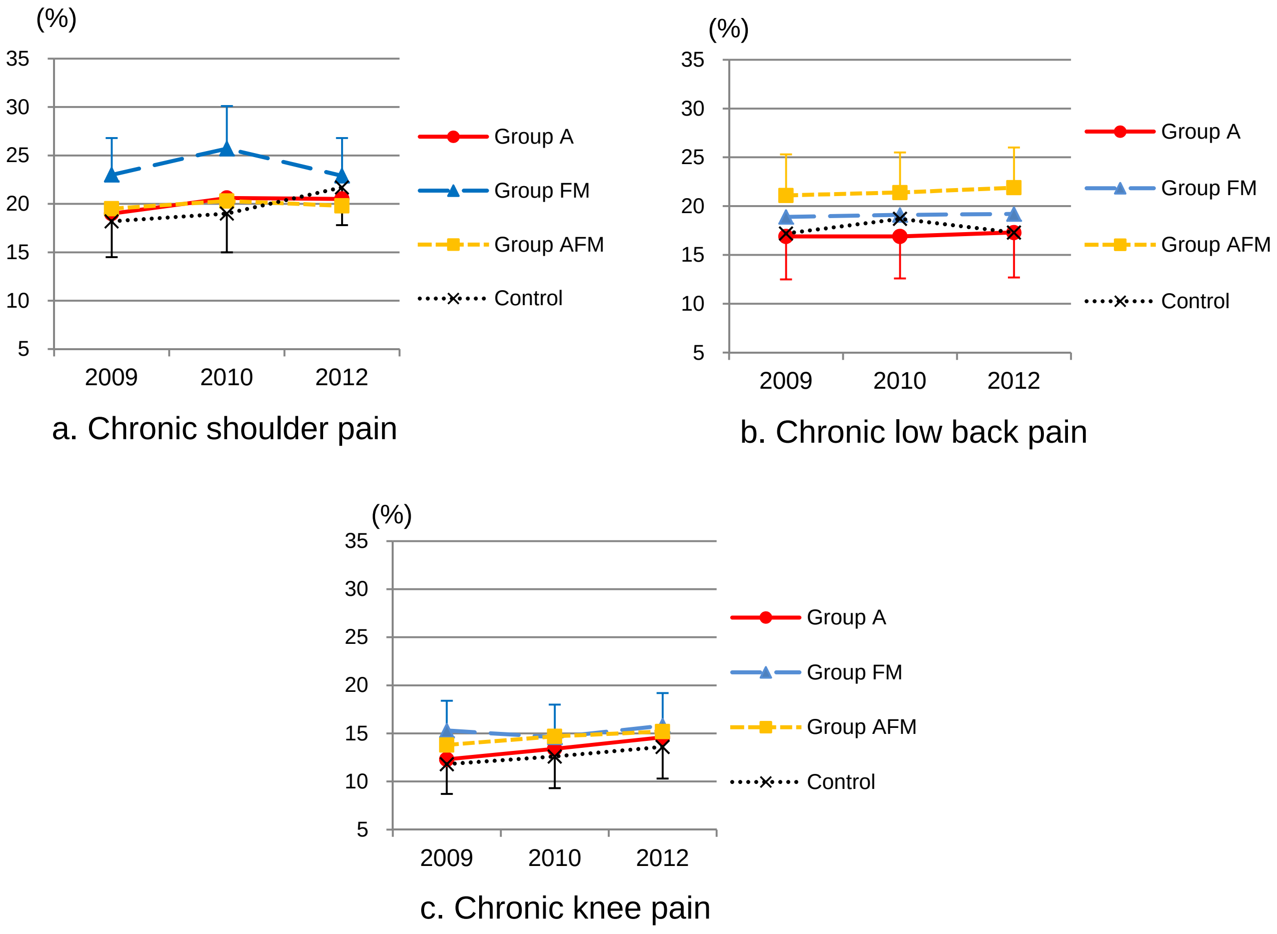

Additional file 6: Figure. Adjusted prevalence of chronic musculoskeletal pain over the 3-year intervention period.

Adjusted prevalence is shown for chronic shoulder (a), low back (b), and knee pain (c). In each community in the intervention subgroups, aerobic activity (Group A), flexibility and muscle-strengthening activities (Group FM), or all aerobic, flexibility, and muscle-strengthening activities (Group AFM) were promoted. The prevalence is adjusted for sex, age, body mass index, self-rated health, years of education, employment status, engagement in farming, baseline overall physical activity, chronic disease history, and community (cluster) where the respondents lived; 95% confidence intervals are presented only for groups with the lowest or highest prevalence over the period. Range of confidence-bar lengths is 3.1–5.5.
